# Quantifying Proteinuria in Hypertensive Disorders of Pregnancy

**DOI:** 10.1155/2014/941408

**Published:** 2014-09-16

**Authors:** Sapna V. Amin, Sireesha Illipilla, Shripad Hebbar, Lavanya Rai, Pratap Kumar, Muralidhar V. Pai

**Affiliations:** Department of Obstetrics and Gynaecology, Kasturba Hospital, Kasturba Medical College, Manipal University, Manipal, Karnataka 576104, India

## Abstract

*Background.* Progressive proteinuria indicates worsening of the condition in hypertensive disorders of pregnancy and hence its quantification guides clinician in decision making and treatment planning. *Objective.* To evaluate the efficacy of spot dipstick analysis and urinary protein-creatinine ratio (UPCR) in hypertensive disease of pregnancy for predicting 24-hour proteinuria. *Subjects and Methods.* A total of 102 patients qualifying inclusion criteria were evaluated with preadmission urine dipstick test and UPCR performed on spot voided sample. After admission, the entire 24-hour urine sample was collected and analysed for daily protein excretion. Dipstick estimation and UPCR were compared to the 24-hour results. *Results.* Seventy-eight patients (76.5%) had significant proteinuria of more than 300 mg/24 h. Dipstick method showed 59% sensitivity and 67% specificity for prediction of significant proteinuria. Area under curve for UPCR was 0.89 (95% CI: 0.83 to 0.95, *P* < 0.001) showing 82% sensitivity and 12.5% false positive rate for cutoff value of 0.45. Higher cutoff values (1.46 and 1.83) predicted heavy proteinuria (2 g and 3 g/24 h, resp.). *Conclusion.* This study suggests that random urinary protein : creatine ratio is a reliable investigation compared to dipstick method to assess proteinuria in hypertensive pregnant women. However, clinical laboratories should standardize the reference values for their setup.

## 1. Introduction

Haemorrhage, sepsis, and hypertension during pregnancy are the important cause for maternal morbidity and mortality in India and worldwide [1]. Among hypertensive disorders of pregnancy preeclampsia is the leading cause and complicates 5%–10% of pregnancy. Preeclampsia is a multisystem disorder characterized by reduced renal perfusion and damage to glomerular basement membrane resulting in leakage of proteins in urine. Irrespective of the cause of hypertension, quantification of proteinuria in the pregnancy is important not only for making diagnosis, but also for predicting maternal and fetal outcome. Normal women excrete minimal quantity of proteins in the urine (up to 150 mg/day), but because of renal changes that occur during pregnancy, proteinuria in excess of 300 mg/day is considered as abnormal for pregnant women. The methods to quantify proteinuria vary, but till today 24-hour urine protein measurement is considered as gold standard for protein estimation [2].

However the 24-hour urine protein excretion method is cumbersome and requires admission and it is costly and time consuming and its usefulness is limited to collection errors, storage difficulties, specimen handling, and poor patient compliance. Not only there is a delay in diagnosis due to waiting time, but also this method proves pointless when urgent delivery is required due to worsening maternal and foetal condition. Considering these issues, alternative methods for diagnosis of proteinuria in pregnancy have been thought off, which include dipstick method and spot urinary protein : creatinine ratio. The dipstick is inexpensive, easy to use, and rapid and the test can be done by paramedical health assistants including the patient herself. But their usefulness is limited due to their low sensitivity and specificity [3–5]. More so ever, the inherent inaccuracies in dipstick analysis are thought to be influenced by maternal hydration status, diurnal variation of protein excretion, orthostatic proteinuria, exercise, presence of infection, and other contaminants in the urine such as phosphates.

Of late, spot urine protein : creatinine ratio (UPCR) has been demonstrated to correlate well with 24 hours proteinuria in patients with renal diseases, such as membranous and proliferative glomerulonephritis, diabetic nephropathy, lupus nephritis, and renal transplants [6–9]. The diurnal variation of specific gravity of urine due to changing glomerular filtration rate (GFR) results in varying concentrations of urinary protein at different times of the day. But when this concentration is divided by spot urinary creatinine level which is GFR dependent, it results in a constant ratio throughout the day and, hence, is considered to be a reliable indicator of proteinuria. The spot urine protein : creatinine ratio can be ordered on outpatient basis, the results are available in a short time and suppose to help the obstetrician in quick decision making and management planning.

The present study aims at comparison of diagnostic utility of two tests: urine dipstick method and spot urine protein : creatinine ratio in diagnosis of significant proteinuria in patients with hypertensive disorder of pregnancy for our hospital, which is a teaching hospital for Kasturba Medical College, Manipal, which caters to the need for more than six districts of Karnataka state, representing South Indian population. The secondary objective is to establish UPCR reference standards for our setting for not only proteinuria of ≥300 mg/24 hours, but also for prediction of higher degree of proteinuria (2 g and 3 g/24 h, resp.) which may help obstetrician to plan timing of delivery in severe preeclampsia.

## 2. Materials and Methods

The study was conducted in the Department of Obstetrics and Gynaecology, Kasturba Medical College, Manipal University, Manipal, India for 2 years (July 2009 to June 2011). Institutional ethical committee approval was obtained before the start of the study. A total of 102 patients who had hypertensive disorders of the pregnancy were recruited after 20 weeks of gestation and these patients had detailed medical and obstetrical history, general physical & systemic examinations, and other investigations required for the management.

Exclusion criteria included all cases of chronic renal disease, secondary hypertension due to immunological diseases such as lupus erythematosus, and overt diabetes mellitus. Patients who were delivered due to urgent indications for termination of pregnancy and hence could not complete 24-hour collection were also excluded.

Hypertension was diagnosed when diastolic pressure exceeded 90 mm Hg or more on two occasions four hours apart, a single recording of 110 mm or an increase in systolic blood pressure by 30 mm Hg, and diastolic 15 mm Hg above previously recorded blood pressure readings. Gestational hypertension was defined as systolic blood pressure ≥140 mg and/or diastolic blood pressure ≥90 mg/dL in a previously normotensive pregnant woman after 20 weeks of gestation without proteinuria or a sign of end-organ dysfunction and whenever in addition proteinuria was present; patient was diagnosed to have preeclampsia.

### 2.1. Sample Size Determination

Calculated sensitivity of the urine protein : creatinine ratio for detecting significant proteinuria is around 72% (Aggarwal et al. [10] from PGIMER, Chandighar). The minimum required sample size is determined by Buderer's formula for sensitivity and specificity studies;

(1) $$\begin{array}{c} N=\frac{\left[Z_{1-\alpha /2}^{2}\times P\times \left(1-P\right)\right]}{L^{2}}, \end{array}$$

where in, *N*  = number of patients, *Z*_1−*α*/2_ = 1.96 (standard normal deviate value that divides the central 95% of *z* distribution from 5% in the tails), *P*  = the reported sensitivity (72%, i.e., 0.72), and *L*  = absolute precision desired on either side (half width of the confidence interval of the confidence interval) of sensitivity (10%, i.e., 0.1).

Accordingly sample size required is 78 and we have 102 subjects in the present study which is more than minimum required.

### 2.2. Urine Dipstick Test

Patient was asked to submit random midstream urine sample prior to admission in a 50 mL urine container for laboratory analysis for random urine dipstick test, protein, and creatinine. The dipstick analysis was done using the uriplus 900 urinalysis stip. The following are the grades of proteinuria as provided by the manufacturers:

 0: absent
 Traces: 15 to 30 mg/dL
 1+: 30 to 100 mg/dL
 2+: 100 to 300 mg/dL
 3+: 300 to 1000 mg/dL
 4+: greater than 1000 mg/dL.

### 2.3. Random Urine Protein Estimation

The random urine protein and creatinine estimations were performed on the same sample of urine which was mentioned previously. Urinary total protein was analysed using Turbidimetric method with benzethonium chloride precipitation as describe by Iwata and Nishikaze. Initially the urine was mixed with buffer solution containing diluted NaOH to chelate nonprotein components such as calcium and magnesium which interfere with the protein determination and also to make the solution alkaline. Baseline absorbance was measured at 660 nm and then benzethonium chloride, a quaternary ammonium salt, is added. The protein reacts with benzethonium and produces a turbidity that is very stable and less dependent on temperature. The absorbance was remeasured after 10 minutes and the difference in absorbance indicated the protein concentration. The whole process was automated (COBAS 6000 system).

### 2.4. Creatinine Estimation

Urinary creatinine measurement was carried out on the same random urine sample. Estimation of urine creatinine was based upon the principle of Jaffe's reaction that at alkaline pH, creatine reacts with picric acid to produce creatinine alkaline picrate which gives orange colour to the solution. The magnitude of this change can be measured at a wave length of 492 nm with a colourimeter (inbuilt in auto analyzer) after incubating the solution at 37°C and the concentration of urinary creatinine can be obtained by calibrating against a solution of known creatinine concentration.

### 2.5. Random Urinary Protein : Creatinine Ratio

After obtaining the both random urine protein and creatinine concentrations in mg per 100 mL, ratio was calculated by simply dividing protein concentration by creatinine concentration.

### 2.6. 24-Hour Urine Protein Estimation

24-hour urine protein estimation was carried out after admission. A previous urine microscopic examination ruled out urinary tract infection which otherwise would interfere with protein estimation. Patient was asked to discard the first void early morning sample. Thereafter from the same time till next 24 hours, patient was instructed to collect all the voided samples in a large container which was sent to laboratory for further analysis. The adequacy of 24-hour urine collection was cross-checked with creatinine in the sample to the predicted creatinine concentrations as estimated by the Cock Croft Gault equation for women. The urine was stirred to get a homogenous sample. Urine protein estimation was carried out in the same manner that is described for random sample on 4 mL aliquot and expressed as mg/dL. 24-hour urine protein was estimated by the formula

(2) Total  24  hour  urine  protein  excretion =Urine  protein  concentration  (mg/dL)  ×24  hour  urine  volume  in  mL/100.

### 2.7. Statistical Analysis

SPSS software (version 16, Chicago II, USA) was used to arrive at statistical inference. Descriptive statistics were used to calculate mean, standard deviation, and minimum and maximum values. Ordinal variables were analysed using cross tabulation. Chi square test was performed to test ability of urine dipstick method to quantify proteinuria. *P* value of <0.05 was considered significant. Receiver operator characteristic (ROC) analysis was carried out to determine the best cutoff values for urinary protein : creatinine ratio (UPCR) to detect proteinuria range of 300 and 3000 mg. ROC curves were drawn using Microsoft Excel 2010 for sensitivity and specificity values generated by SPSS program. Areas under ROC curves (AUC) along with their 95% confidence intervals were compared to determine diagnostic abilities of these two methods to detect significant proteinuria. Three statistical ratios (positive likelihood ratio, negative likelihood ratio, and odd's ratio) were computed and compared with each other for respective best determined test cutoff values. Pearson correlation coefficient was used to assess relationship between 24-hour urine protein and urine protein : creatinine ratio.

## 3. Results

Demographic profile of the study subjects is shown in Table 1. Both primigravidae and multigravidae almost equally contributed for the study (51% and 49%). The majority of patients were in third trimester of pregnancy (92%, 94/102) and 69% (71/102) belonged to gestational age more than 34 weeks. There were only four (3.9%) with chronic hypertension. The incidence of severe preeclampsia (including imminent eclampsia) was relatively high (76.5%, 78/102) as our hospital is one of the tertiary referral center for surrounding four districts. 48% (49/102) had preterm birth and the majority had caesarean delivery (72.5%, 74/102). Table 1 also describes maternal, foetal, and neonatal complications with numbers and percentages.

The laboratory parameters are shown in Table 2. The total 24 hours urine volume ranged from 1350 to 2980 mL. The mean and standard deviation of total protein excretion per day were 1446 mg and 1242 mg, respectively, (minimum 112 mg/day and maximum 4850 mg/day). Percentage distribution for different urine dipstick values (ranging from absent to 4+ proteinuria) is classified in Table 2. Less than 1+ proteinuria (47%, 48/102) indicated negligible proteinuria by dipstick standards, whereas when 24-hour urine protein estimation was done, 24 subjects (23.5%) had insignificant proteinuria of less than 300 mg/day which is a defined cutoff pregnant women. 76.5% (78/102) had significant proteinuria and of them 66 (64.7%) had proteinuria ranging between 300 mg and 3000 mg/day. Only 12 (11.8%) had more than 3 grams proteinuria in our study. The mean ± standard deviation and range of values for random urine protein & creatinine spot urine protein : creatinine ratio (UPCR) are given Table 2.

Table 3 describes diagnostic accuracy of urine dipstick test to detect proteinuria in preeclampsia patients at various grades. 1+ was found to be best cutoff to detect 300 mg of protein excretion per day with sensitivity and specificity of 59% and 66.7%. At other cutoffs specificity improves, but sensitivity is compromised. Linear relationship analysis between different dipstick values and 24-hour total protein excretion showed regression coefficient (*R* ^2^) 0.33, which indicated poor relationship (value close to 1 indicate strong relationship).

**Figure 1:**
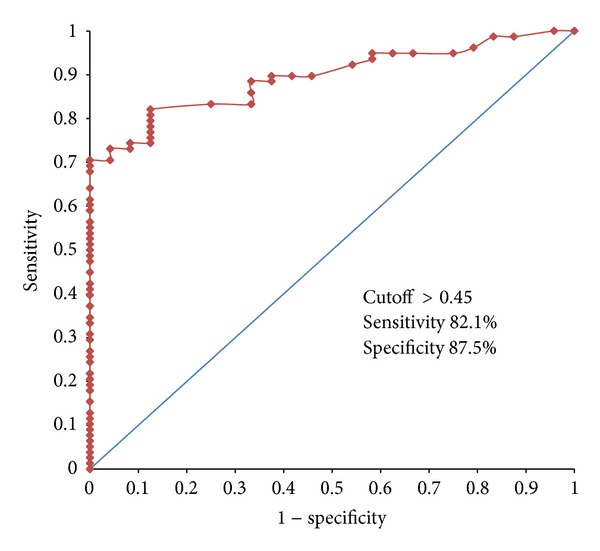
ROC curve for urine protein : creatinine ratio to predict proteinuria of 300 mg/24 h.

The diagnostic utility of a test is usually derived by ROC analysis which enables determination of sensitivity, specificity, and other parameters at different cutoff values.  Figure 1 indicates ROC curve for urine protein : creatinine ratio to predict 300 mg proteinuria/24 hours. The area under the curve (AUC) component of ROC graph is an indicator of discriminatory power of the test, which when approaches 1.0, will give almost 100% sensitivity and specificity. This value was calculated as 0.89 (95% CI 0.83–0.95), which was statistically significant (*P* < 0.001).  Table 4 indicates test performances at five different cutoffs based on ROC findings. It can be seen that urine protein : creatinine ratio of 0.45 had the best combination for maximum sensitivity, specificity, and predictive values. Though other cutoff values show high percentage for some parameters, they perform poorly in other aspects.

**Figure 2:**
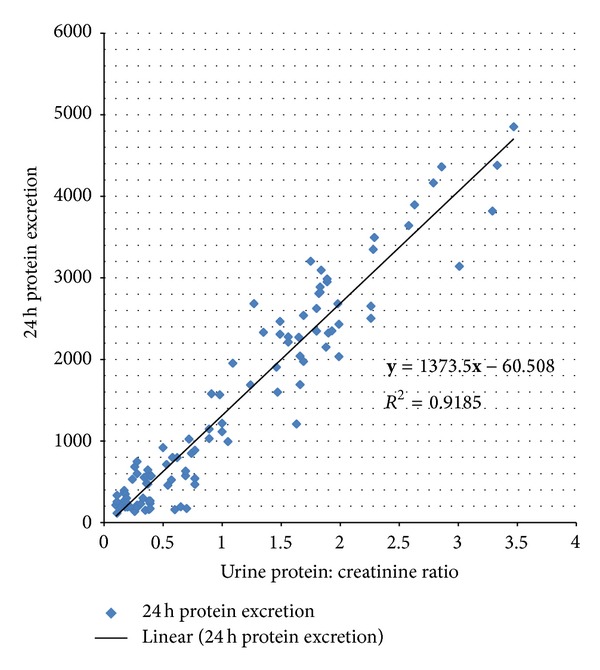
Relationship between urine protein : creatinine ratio and 24 hours proteinuria.

Figure 2 indicates linear strong relationship between 24 hours urine protein excretion and urine protein : creatinine ratio (coefficient of determination *R* ^2^ = 0.9185, *F*-ratio 1127.69, significance level *P* < 0.001). The predicted daily urinary excretion in mg (**y**) based on spot urine protein : creatinine ratio (**x**) is given by the equation

(3) $$\begin{array}{c} y=-60.5083+1373.4529x. \end{array}$$

Table 5 shows ability of urine protein : creatinine ratio to predict 24 hours proteinuria of (more than 2000 mg and 3000 mg per day) in comparison to 300 mg proteinuria. An UPCR value of 1.46 had good test characteristics for prediction of 2 grams proteinuria with good likelihood ratios (both LR+ and LR−). The value for LR+ for this cutoff was 12.5 (in general LR+ >10 is supposed to change pretest probability to higher posttest probability and is considered as strong diagnostic test). This would mean that pretest probability of 35% (36 out of 102 our subjects had proteinuria more than 3 grams/day) would indicate 99% chance of having proteinuria of >3 gms/day, if UPCR is ≥1.46. Though an UPCR cutoff of 1.83 had good sensitivity and specificity of detection of 3 grams+ proteinuria, the positive predictive value was only 47.8%. This is because there were only 11.8% (12/102) of patients in our series which had proteinuria of more than 3 grams per day.

## 4. Discussion

Since time immortal, urine examination remains one of the important examinations during antenatal checkups. The appearance of proteins in the urine heralds possible onset of hypertensive complication, either proteinuric gestational hypertension or superimposed preeclampsia over preexisting renal disease. The quantity of protein loss has both diagnostic and prognostic implications, but what constitutes an ideal test still remains controversial.

Three methods of urine protein estimation have been used amply in the current obstetric practice. The most popular one is urine dipstick analysis which is readily available in most of the hospitals and is also semiquantitative, the second one is so called “gold standard” 24 hours urinary proteins but is limited by its availability and time constraints, and the third one is slowly becoming popular, that is, the estimation of ratio of either protein or albumin to the creatinine concentration (urinary protein : creatine ratio (UPCR) and urine albumin : creatinine ratio (UACR)) in the random urine sample. This method gives faster and reasonably accurate assessment of significant proteinuria. Of the two, the first one is preferred as the second ratio is associated with relatively low sensitivity and high false positivity [25].

The dipstick method is economic and simple to perform [26]. However it is not a recommended test, as studies quote substantial false positive rates, poor sensitivity, and accuracy. Though classically +1 dipstick grade has been considered as a marker of pathological proteinuria (protein excretion of >300 mg/day) in pregnant women, the grading can vary depending upon maternal hydration status. Thus, even trace proteinuria may get reported as significant if mother is dehydrated and vice versa if mother is over hydrated. The grading can alter depending upon lab technician's expertise, alkalinity of urine, and presence of infection. The reported sensitivity for visual urine dipstick test varies widely from 51% to 85% (Table 6), which is 51% in our study. If one uses automated analyser, higher accuracy may be obtained [27]. These analysers are portable reflectance photometers that can be calibrated to read a variety of reagent strips and hence free from subjectivity.

The best way to quantify proteinuria is to measure its daily renal excretion. Nonpregnant women excrete up to 150 mg per day, whereas in pregnancy the cutoff is 300 mg. For 24 h protein estimation, the urine has to be collected at each time of voiding and requires being stored in a large glass container before being sent to the laboratory. The disadvantages are many, to mention some are lack of compliance, inconvenience to patients, hospitalization, and so forth. In addition, rest in supine position during hospital stay may result in stagnation of urine in renal pelvicaliceal system and volume of collected urine may not reflect actual 24 hours secretion. So there are continuous efforts to replace this test with spot tests such as urine protein : creatinine ratio and albumin : creatinine ratio [28]. These ratios are not affected by variations in the concentration of urine and the amount of urine excreted in 24 hours.

Several studies (Table 7) have established the usefulness of urine protein : creatinine ratio not only to predict 300 mg proteinuria, but also to predict the higher range of proteinuria at different cutoffs such as 2 grams, 3 grams, and 5 grams there by guiding the physician to make the diagnosis of severe preeclampsia and thereafter to institute appropriate obstetric management [18]. The cutoff for proteinuria in severe preeclampsia differs from country to country. In China, it is taken as 2.0 g/24 h. in UK it is 3.0 g/24, whereas in the United States, severe preeclampsia by proteinuria is defined as 5.0 g/24 h [8]. A Korean study revealed that the optimal random protein : creatinine ratio cutoff points as 0.63 and 4.68 for 300 mg/24 h and 5.0 g/24 h [11]. In our study, urine protein : creatinine ratio of 0.45, 1.46, and 1.83 predicted 24 h urine protein excretion of 300 mg, 2 g, and 3 g with reasonable sensitivity and specificity (Table 5).

There appears to be very strong linear correlation between urine protein : creatinine ratio and degree of proteinuria [17, 19, 21, 29]. Lower ratios predict lesser degree of proteinuria and higher ones larger degree.

However the cutoff values for urine protein : creatinine ratio differs from center to center from 0.18 to 1.14 [12–16, 20, 22–24]. These differences exist because of variation in patient selection, laboratory methods used to estimate urine protein and creatinine levels (various reagents, manual or automated methods), and importantly appropriateness of urine collection. Inclusion criteria varied from gestational hypertension [14], mild preeclampsia only [5, 13, 15], severe gestational hypertension and preeclampsia [16, 18], early and late onset preeclampsia [10, 23], late onset preeclampsia [11, 22, 24] to any hypertension in pregnancy including chronic hypertension [12, 17, 19–21]. The methods to estimate 24-hour urine varied from one study to other, for example, Biuret method [10, 13, 18, 19, 24], Bradford assay [17], Coomassie reagent method [22], Polyethylene glycol turbidimetry [20], Pyrogallol red reaction [5, 11, 12, 15], Trichloroacetic acid [14, 16], and Turbidimetric method [21] (Table 7). The sensitivity and false positive rates for each type of protein estimation differ significantly [30]. The benzethonium chloride method used in this study has good range of both sensitivity and specificity and is widely used by laboratories all over the world. Though modified Jaffe's two-point rate method is used to estimate urinary creatinine universally, the exact values depend upon whether manual or automated machines are used for its estimation. Similarly adequacy of 24-hour collection is not uniformly reported from all the studies. One study [5] has defined that 24-hour urine collection was satisfactory and complete when it had creatinine of >1000 mg (850 mg for obese women) or a total creatinine of 13 mg per kg; another study stated that urine collection was complete when urine volume per day was more than 1 liter per day with urinary creatinine >1 g/day [18]. Several other studies are of the opinion that adequacy of 24-hour urine collection should be assessed depending upon predicted creatinine excretion by Cock Croft Gault equation for women (should be within 20% range of actual excretion) [17, 21]. It is also important to rule out urinary tract infection to obtain correct values and only few studies have mentioned that samples with evidence of pus cells and bacteruria were discarded [12, 15, 16, 18]. Adhering to strict protocols may result in different cutoff values for UPCR, which is evident in reviewed studies (Table 7).

## 5. Conclusion

Urine protein : creatine ratio is one of the important investigation in hypertensive disorder of the pregnancy. It is simple, accurate, and convenient measurement which is not only qualitative, but also semiquantitative as it can predict the total amount of protein loss through kidneys. It is a good replacement for tedious, time consuming 24 h urine protein estimation, especially in countries like India where hospitals cannot cope up with a large number of in-patients. It can also serve as a useful gadget to monitor proteinuria on outpatient basis in hypertensive pregnant women during their regular antenatal visits. Nevertheless, the diagnostic performance of this ratio is influenced by variability in laboratory methods and hence it is suggested that the reference laboratories should develop their own local standards and cutoff values which are periodically calibrated and prospectively validated.

## Figures and Tables

**Table 1:** Baseline clinical characteristics of study population.

Parameter	Value
Age in years	27.4 ± 4.3 (20–41)
Gestational weeks at delivery	35.3 ± 3.3 (25–39)
Systolic blood pressure at the time of admission (mm Hg)	152 ± 18.2 (132–178)
Diastolic blood pressure at the time of admission (mm Hg)	96.4 ± 11.3 (78–116)
Parity	
Primiparae	52 (51%)
Multiparae	50 (49%)
Type of hypertension	
Chronic hypertension	4 (3.9%)
Gestational hypertension	17 (16.7%)
Mild preeclampsia	22 (21.6%)
Severe preeclampsia	43 (42.2%)
Imminent eclampsia	13 (12.7%)
Eclampsia	3 (2.9%)
Obstetric complications	
Gestational diabetes	9 (8.8%)
Thrombocytopenia	8 (7.8%)
Anemia	2 (2%)
Twins	2 (2%)
Abruptio placenta	2 (2%)
Intrauterine death	3 (2.9%)
HELLP syndrome	4 (3.9%)
Fetal growth restriction	10 (9.8%)
Oligohydramnios	4 (3.9%)
Raised Doppler indices	5 (4.9%)
Preterm delivery	49 (48%)
Route of delivery	
Vaginal delivery	28 (27.5%)
Caesarean delivery	74 (72.5%)
Birth weight (Kg)	
<1 Kg	7 (6.9%)
1–1.5 Kg	16 (15.7%)
1.51–2.5 Kg	32 (31.4%)
>2.5 Kg	47 (46.1%)
Overall	2.16 ± 0.73 (0.56–3.4)
Neonatal complications	
Hyperbilirubinaemia	8 (7.8%)
Respiratory distress	7 (6.9%)
Sepsis	1 (1%)
Still birth	4 (3.9%)
Transient tachypnoea	17 (16.7%)

**Table 2:** Laboratory evaluation.

Parameter	Value
24 hr urine volume (mL)	2232 ± 490 (1350–2980)
Total protein excretion per day (mg)	1446 ± 1242 (112–4850)
Spot urinary protein per dL	50.9 ± 42.7 (10–191)
Urine creatinine per dL	52.8 ± 25.6 (30–190)
Urine protein : creatinine ratio (UPCR)	1.09 ± 0.86 (0.1–3.47)
Urine dipstick	
Absent	19 (18.6%)
Traces (15 to 30 mg/dL)	29 (28.4%)
1 + (30 to 100 mg/dL)	25 (24.5%)
2 + (100 to 300 mg/dL)	12 (11.8%)
3 + (300 to 1000 mg/dL)	10 (9.8%)
4 + (greater than 1000 mg/dL)	7 (6.9%)
Proteinuria range	
Less than 300 mg/day	24 (23.5%)
300–1000 mg/day	28 (27.5%)
1001–2000 mg/day	14 (13.7%)
2001–3000 mg/day	24 (23.5%)
More than 3000 mg/day	12 (11.8%)

**Table 3:** Diagnostic test characteristics at different dipstick grades to predict proteinuria of 300 mg/day or more.

Cutoff values	1+	2+	3+	4+
Sensitivity (%)	59	37.2	21.8	22.6
Specificity (%)	66.7	100	100	100
PPV (%)	85.2	100	100	100
NPV (%)	33.3	32.9	28.2	50
Accuracy (%)	60.8	52	40.2	56.4
Positive likelihood ratio (LR+)^#^	1.77	18.22	10.68	11.06
Negative likelihood ratio (LR−)^#^	0.62	0.64	0.8	0.79
Odd's ratio^#^	2.88	28.41	13.38	14

^#^0.5 was added to empty cells to calculate ratios.

**Table 4:** Diagnostic test characteristics at various cutoff values for UPCR to predict proteinuria of 300 mg/day or more.

Cutoff values	0.30	0.45	0.60	0.75	0.90
Sensitivity (%)	89.7	82.1	75.6	67.9	61.5
Specificity (%)	54.2	87.5	87.5	100.0	100.0
PPV (%)	86.4	95.5	95.2	100.0	100.0
NPV (%)	61.9	60.0	52.5	49.0	44.4
Accuracy (%)	81.4	83.3	78.4	75.5	70.6
Positive likelihood ratio (LR+)^#^	1.96	6.56	6.05	33.29	30.15
Negative likelihood ratio (LR−)^#^	0.19	0.21	0.28	0.32	0.38
Odd's ratio^#^	10.34	32	21.74	101.76	76.8

^#^0.5 was added to empty cells to calculate ratios.

**Table 5:** Diagnostic ability of urine protein : creatinine Ratio (UPCR) for various proteinuria range.

Proteinuria range	Cutoff values	Sensitivity (%)	Specificity (%)	PPV (%)	NPV (%)	Accuracy (%)	Positive likelihood ratio (LR+)	Negative likelihood ratio (LR−)	Odd's ratio	Area under curve & 95% CI
UPCR to predict 300 mg+/day	0.45	82.1	87.5	95.5	60.0	83.3	6.6	0.21	32	0.89 (0.83–0.95)
UPCR to predict 2000 mg+/day	1.46	94.4	92.4	87.2	96.8	93.1	12.5	0.06	207	0.98 (0.97–100)
UPCR to predict 3000 mg+/day	1.83	91.7	86.7	47.8	98.7	87.3	6.9	0.09	6.88	0.98 (0.94–100)

**Table 6:** Diagnostic utility of urine dipstick method for detection of significant proteinuria.

Study	Urine dipstick value	Sensitivity (%)	Specificity (%)	Area under receiver operating curve (95% CI)	Positive likelihood ratio	Negative likelihood ratio
Waugh et al. 2005 [4]	1+	51	78	∗	2.27	0.635
Dwyer et al. 2008 [5]	1+	41	100	0.71 (0.64–0.77)	#	0.59
Park et al. 2013 [11]	1+	85	95	0.93 (0.88–0.99)	17	0.15
Present study 2014	1+	59	66.7	0.66 (0.54–76)	1.42	0.34

∗ not calculated in the study.

# indicates very high values, but cannot be calculated because of empty cell in 2 × 2 table.

**Table 7:** Reported cutoff values for UPCR and diagnostic summary in pregnant women with hypertensive disorders.

Study	UPCR	Protein estimation method	Sensitivity (%)	Specificity (%)	Area under receiver operating curve (95% CI)	Positive likelihood ratio	Negative likelihood ratio
Rodriguez-Thompson and Lieberman 2001 [12]	0.19	Pyrogallol red reaction	90	70	0.91 (0.87 to 0.96)	3	0.14
Durnwald and Mercer 2003 [13]	0.39	Biuret method	72.6	73.1	0.8	2.7	0.37
Al et al. 2004 [14]	0.19	Trichloroacetic acid	85	73	0.86 (0.80 to 0.93)	3.15	0.21
Yamasmit et al. 2004 [15]	0.25	Pyrogallol red reaction	96.6	92.3	0.93	12.55	0.04
Taherian et al. 2006 [16]	0.18	Trichloroacetic acid	86.3	100	0.94 (0.88 to 0.98)	#	0.14
Leaños-Miranda et al. 2007 [17]	0.30	Bradford method	98.2	98.8	∗	81.8	0.02
Wheeler et al. 2007 [18]	0.21	Biuret method	86.8	77.6	0.82	3.88	0.17
Aggarwal et al. 2008 [10]	1.14	Biuret method	72	75	∗	2.88	0.37
Shaikh et al. 2010 [19]	0.20	Biuret method	91.2	87.8	0.84	7.47	0.10
Dwyer et al. 2008 [5]	0.28	Pyrogallol red reaction	65	95	0.83 (0.76–0.88)	13	0.36
Kyle et al. 2008 [20]	0.27	Polyethylene glycol turbidimetry	92.3	97.1	∗	31.82	0.07
Eslamian et al. 2011 [21]	0.22	Turbidimetric method	87	92.6	0.93 (0.85–0.99)	11.76	0.14
Kumari et al. 2013 [22]	0.30	Coomassie reagent method	90	84	0.83	5.65	0.12
Gaddy-Dubac et al. 2013 [23]	0.30	Not mentioned	32.9	85	0.74 (0.67–0.82)	2.19	0.79
Sharma et al. 2013 [24]	0.25	Biuret method	69	75	0.79 (0.66–0.92)	2.76	0.41
Park et al. 2013 [11]	0.63	Pyrogallol red reaction	87.1	100	0.956 (0.90–1.00)	#	0.13
Present study 2014	0.45	Benzethonium method	82.1	87.5	0.89 (0.83–0.95)	6.56	0.21

∗ not calculated in the study.

# indicates very high values, but cannot be calculated because of empty cell in 2 × 2 table.
